# *Lactobacillus plantarum* SAL delays aging-associated oxidative stress and gut microbiota dysbiosis in mice

**DOI:** 10.3389/fmicb.2025.1607824

**Published:** 2025-06-16

**Authors:** Wen Dong, Yongzhi Lun, Jie Sun, Ben Liu

**Affiliations:** ^1^Department of Laboratory Medicine, Pharmaceutical and Medical Technology College, Putian University, Putian, China; ^2^Key Laboratory of Screening and Control of Infectious Diseases, Fujian Provincial University, Quanzhou Medical College, Quanzhou, China

**Keywords:** aging, SAL strain, *Lactobacillus plantarum*, oxidative stress, gut microbiota

## Abstract

**Introduction:**

*Lactobacillus plantarum* SAL, isolated from multidrug-resistant patients’ feces, exhibits superior in vitro probiotic traits including bile salt resistance, gastric acid tolerance, and potent antioxidant capacity. While *Lactobacillus plantarum* generally enhances gut microbiota structure/function, improving health and lifespan in model organisms, the in vivo effects, mechanisms, and potential anti-aging properties of the SAL strain remain unexplored. This study addresses this critical research gap.

**Methods:**

Twenty-four SPF KM male mice were divided into a control group (CON), model group (MOD), and a SAL strain intervention group (SAL). MOD and SAL groups received d-gal-induced aging models. SAL group was orally administered SAL strain suspension daily, while MOD and CON groups received saline for 10 weeks. After the intervention, serum and liver tissues were collected to detect aging biomarkers (β-galactosidase) and oxidative stress markers.Colon tissue histopathological examination was performed, and fresh fecal samples were subjected to metagenomic sequencing and analysis. Additionally, Spearman correlation analysis was conducted to evaluate the relationships between genuslevel differential gut microbiota and oxidative stress markers in serum and liver tissues.

**Results:**

Compared with the MOD group, the SAL group exhibited significantly reduced MDA levels in serum and liver tissues (all *p* < 0.05), elevated activities of SOD and T-AOC (all p < 0.05), and increased serum GSH-Px and CAT activities (all *p* < 0.05). Colon histology showed structural improvements, including increased crypt numbers, restored architecture, reduced submucosal space, and upregulated expression of *ZO-1*, *Occludin*, and *Muc2* (all *p* < 0.05). Gut microbiota analysis revealed increased abundances of Firmicutes and Verrucomicrobia, decreased Bacteroidetes, and elevated Firmicutes/Bacteroidetes (F/B) ratio (*p* < 0.05). Differential genera *Lactobacillus* and *Mucispirillum* showed significant negative correlations with MDA levels (all *p* < 0.05), while *Lactobacillus* positively correlated with SOD, GSH-Px, and T-AOC activities.

**Discussion:**

The SAL strain intervention significantly improved redox homeostasis, restored intestinal barrier integrity, and reversed gut dysbiosis, highlighting its dual regulatory role in anti-aging mechanisms. These findings demonstrate the potential of *L. plantarum* SAL as an anti-aging probiotic and establish a theoretical framework for microbiota - targeted interventions to alleviate age-related pathologies.

## 1 Introduction

Aging, an irreversible biological process underlying chronic disease development, poses growing challenges as global populations age. Rising rates of cardiovascular disorders, neurodegenerative conditions, and diabetes strain healthcare systems and reduce life quality (Leton, 2024). Oxidative stress—characterized by free radical accumulation exceeding antioxidant defenses—drives cellular dysfunction and multi-organ degeneration (Maldonado et al., 2023; Santos et al., 2024). Emerging research highlights the gut microbiota’s dual role in aging through microbial diversity loss (Escudero-Bautista et al., 2024; Li and Roy, 2023) and redox balance regulation. Age-related dysbiosis impairs intestinal integrity and metabolic homeostasis, accelerating aging processes (Beaver et al., 2025; He et al., 2024). Targeted microbial modulation presents a promising therapeutic strategy to concurrently address oxidative damage and fundamental aging mechanisms, offering potential solutions for aging-related health burdens.

*Lactobacillus plantarum* is ubiquitous in nature, particularly in various fermented foods and the intestines of animals. It is a common probiotic known for its multiple significant biological properties and health benefits, such as antioxidant, immunomodulatory, and gut microbiota-modulating effects (Kumar H. et al., 2022; Park et al., 2020). Numerous studies have demonstrated that the consumption of *L. plantarum* can enhance the structure and function of the gut microbiota, thereby improving the health of model organisms and extending their lifespan (Jin et al., 2024; Song et al., 2022). As a potential anti-aging probiotic, *L. plantarum* has garnered considerable attention.

The *L. plantarum* SAL, isolated from multidrug-resistant patients’ fecal samples by the research team led by Yongzhi Lun, demonstrates exceptional probiotic potential. Genomic characterization reveals robust carbohydrate metabolism pathways coupled with antimicrobial activity and environmental stress tolerance. While virulence-associated genes related to immune evasion and adhesion were identified, phylogenetic analysis classifies the SAL strain as a non-pathogenic variant with attenuated virulence. *In vitro* assessments confirm superior probiotic traits, including bile salt resistance, gastric acid tolerance, and potent antioxidant capacity (Sun et al., 2020). However, research on the *in vivo* effects and mechanisms of the SAL strain is limited, and whether it possesses anti-aging effects remains unexplored.

This study employs a d-galactose-induced murine aging model to investigate the capacity of SAL strain intervention in mitigating oxidative stress and modulating gut microbiota dynamics in aging hosts, systematically evaluating the therapeutic effects of the SAL strain. These findings are anticipated to elucidate the dual regulatory effects of the SAL strain on redox homeostasis and microbial ecology, thereby establishing a theoretical foundation for developing microbiota-targeted interventions against age-related physiological decline.

## 2 Materials and methods

### 2.1 Preparation of *Lactobacillus plantarum* SAL

The SAL strain was isolated from fecal samples of patients with potential multidrug resistance and has been preserved at the China General Microbiological Culture Collection Center (CGMCC No. 21786). The SAL strain suspension was prepared as follows: The SAL strain was initially inoculated onto MRS solid medium (Hopebio, China). Single colonies were selected and transferred to MRS broth medium, followed by anaerobic cultivation at 37°C for 24 h. The resulting liquid bacterial culture was centrifuged at 3000 rpm for 20 min to obtain a bacterial pellet. This pellet was then resuspended in distilled water to achieve a *Lactobacillus* suspension with a concentration of 1 × 10^9^ CFU/mL.

### 2.2 Experimental design and sample collection

**Figure 1:**
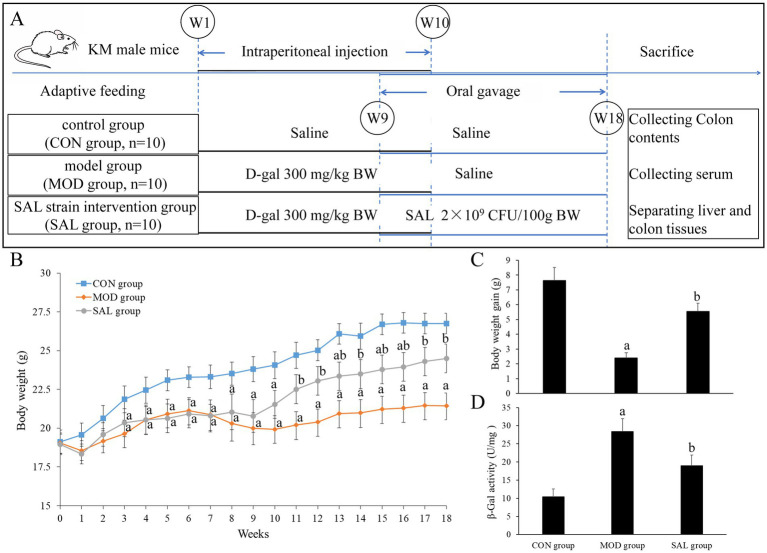
Effect of SAL strain intervention on body weight and β-Gal activity in liver of d-gal-induced aging mice. **(A)** Animal experiment procedure. **(B)** Curves of body weight changes. **(C)** Body weight gain of mice. **(D)** β-Gal activity in the liver. a: Compared with the CON group, *p* < 0.05; b: compared with the MOD group, *p* < 0.05.

Specific pathogen-free (SPF) male KM mice (5–6 weeks old, 20 ± 2 g) were purchased from Shanghai SLAC Laboratory Animal Co., Ltd. (China). A total of 24 mice were housed in a controlled environment with a room temperature of (23 ± 2) °C, relative humidity of (55 ± 10) %, and a 12-h light–dark cycle. All animals had ad libitum access to autoclaved standard chow and UV-sterilized water. After 1 week of adaptive feeding, the mice were randomly assigned into three groups (*n* = 8/group). The groups included a control group (CON group), a model group (MOD group), and a SAL strain intervention group (SAL group). The experimental procedure is shown in Figure 1A. All experiments were conducted in accordance with the *Guide for the Care and Use of Laboratory Animals: Eighth Edition* (ISBN-10: 0–309-153964), and were reviewed and approved by the Animal Ethics Committee of Putian University [Approval No.: 2021 (135)].

To establish the aging model, mice in the MOD group and SAL group were intraperitoneally injected with d-gal (BioDuly, china; 300 mg/kg body weight in 10% w/v sterile saline) daily for 10 weeks. In contrast, mice in the CON group received an equivalent volume of saline for the same period. Starting from the 9th week of model preparation, mice in the SAL group were administered the prepared SAL strain suspension (0.2 mL/10 g body weight, 1 × 10^9^ CFU/mL) via oral gavage daily. Mice in the MOD group and CON group received an equivalent volume of saline during this period. This intervention continued for 10 weeks (total experimental duration: 18 weeks). Throughout this period, body weight and health status (including coat condition, mental status, presence of constipation and other relevant indicators) of all mice were closely monitored and recorded.

At the end of the experiment, the mice were fasted for 12 h with free access to water. Blood was then collected from the abdominal aorta under anesthesia. The Serum was separated and stored at −80°C for subsequent biochemical analysis. Liver tissues and Colon contents were harvested and stored at −80°C for subsequent analysis. The Colon tissues were divided into two parts: the proximal colon was dissected and stored at −80°C for further molecular analyses, while the distal colon was fixed in 4% paraformaldehyde for subsequent histological examination.

### 2.3 Histopathological examination

After being fixed in 4% paraformaldehyde, the colon tissues were embedded in paraffin, sectioned, and stained with hematoxylin and eosin (HE; Solarbio, China) following manufacturer’s protocol. The stained sections were mounted with neutral resin and observed under an optical microscope (Olympus, Japan) to assess morphological changes.

### 2.4 Serum and liver biochemical analysis

The liver tissues were homogenized in ice-cold phosphate-buffered saline (PBS). The homogenates were then centrifuged, and protein concentration in the supernatant was quantified using a BCA protein concentration assay kit (Nanjing Jiancheng Bioengineering Research Institute, China) following manufacturer’s protocol. Subsequently, the contents of malondialdehyde (MDA) and the activities of superoxide dismutase (SOD), glutathione peroxidase (GSH-Px), catalase (CAT), total antioxidant capacity (T-AOC), and β-galactosidase (β-Gal) were measured in both serum and liver tissue homogenate supernatants using commercial kits. The β-Gal kit was purchased from Beijing Solarbio Technology Co., Ltd. (Beijing, China) while the MDA, SOD, GSH-Px, CAT, and T-AOC kits were obtained from Nanjing Jiancheng Bioengineering Research Institute Co., Ltd. (Nanjing, China). All assays were performed according to the manufacturers’ instructions, and data were quantified accordingly.

### 2.5 Gene expression analysis

Total RNA was extracted from colon tissues using Trizol reagent (Takara, China). Following the protocol provided with the reverse transcription kit (Invitrogen, United States), RNA was reverse-transcribed into cDNA. Subsequently, quantitative PCR amplification was performed using a real-time fluorescent quantitative PCR instrument (Bio-Rad, United States). The primer sequences used in this study are detailed in Table 1. For each sample, three technical replicates were set up, with GAPDH serving as the internal reference gene. The average Ct values from these replicates were calculated, and the relative expression levels of the target genes were determined using the 2^−ΔΔCt^ method.

**Table 1:** Oligonucleotide primers used for qRT-PCR.

Gene	Forward	Reverse
*ZO-1*	GCTGGAGAAGATGGAGAA	CAGGTCCTCCTGGTCTTCTC
*Occludin*	CCTCTGGCT TTGCTTCTGTC	TGAGGATGGTGCTGAGTTTG
*Muc2*	GCTGCTCCTGCTGCTACTAC	CAGGTCATCGTCATCGTCTC
*GAPDH*	AGGTCGGTGTGAACGGATTTG	TGTAGACCATGTAGTTGAGGTCA

### 2.6 Western blot analysis

Colon tissues were homogenized in RIPA buffer and centrifuged at 4°C. The protein concentration in the supernatant was determined using a BCA protein concentration assay kit. Subsequently, proteins were separated by SDS-PAGE and transferred onto PVDF membranes. The membranes were blocked with 5% skim milk for 1.5 h at room temperature, followed by incubation with primary antibodies against ZO-1 (Abcam, United States), Occludin (Abcam, United States), and Muc2 (Abcam, United States) overnight at 4°C. Afterward, the membranes were incubated with secondary antibodies (ZSGB-BIO, China) for 1 h at room temperature. Protein bands were visualized using an ECL chemiluminescence kit (Beyotime, China). The relative expression levels of target proteins were quantified by normalizing their band intensities to that of GAPDH (ZSGB-BIO, China), which served as the internal reference protein.

### 2.7 Gut microbiota detection and analysis

DNA was extracted from Colon contents using the cetyltrimethylammonium bromide (CTAB) method. After purification and identification, qualified DNA was fragmented by Covaris M220 ultrasonication (Covaris S2 System, United States). Subsequently, Sequencing libraries were constructed and subjected to stringent quality control procedures. The qualified libraries were then sequenced using the Illumina NovaSeq 6,000 high-throughput sequencing platform (Illumina, United States) to generate a comprehensive dataset. Bioinformatics analyses, including taxonomic profiling using Kraken 2 with Bracken abundance estimation, were conducted to assess the species composition and differences. α-diversity metrics (Observed species, Chao1, Shannon, Simpson) and β-diversity patterns (principal coordinate analysis, PCoA) were computed through QIIME2, while Spearman rank correlations were implemented in R software packages (R Project for Statistical Computing, Austria). All related work, such as DNA extraction, library preparation, sequencing, and bioinformatics analysis, was completed by Shenzhen Microbiome Biotechnology Co., Ltd. (Guangdong, China).

### 2.8 Statistical methods

Data processing and analysis were performed using SPSS 19.0 statistical software (IBM, United States). For data following a normal distribution, results were presented as mean ± standard deviation (S. D.), and one-way ANOVA was used for comparisons among multiple groups, with SNK-q test for pairwise comparisons. For data not conforming to a normal distribution, results were expressed as median [M (P_25_, P_75_)], and the Kruskal–Wallis test was used for comparisons among multiple groups, with Dunn’s test employed for pairwise comparisons. Statistical significance was defined at *p* < 0.05 for all analyses.

## 3 Results

### 3.1 Effect of SAL strain intervention on the body weight of d-gal-induced aging mice

As shown in Figure 1B, starting from the 5th week, the body weight of mice in both the MOD and SAL groups was significantly lower compared with that of the CON group (both *p* < 0.05). From the 10th week, the body weight of mice in the SAL group showed an increasing trend and was subsequently higher compared with that of the MOD group (*p* < 0.05). As shown in Figure 1C, compared with the CON group, mice in the MOD group displayed a lower body weight gain (*p* < 0.05). Additionally, compared with the MOD group, mice in the SAL group exhibited greater body weight gain (*p* < 0.05). These findings indicate that the body weight of d-gal-induced aging mice tended to decrease; however, the supplementation with the SAL strain effectively promoted body weight gain over time.

### 3.2 Effect of SAL strain intervention on β-gal activity in the liver of d-gal-induced aging mice

As shown in Figure 1D, compared with the CON group, the β-Gal activity in the liver of both the MOD and SAL groups increased significantly (*p* < 0.05). This indicates that d-gal-induced aging led to elevated β-Gal activity. Furthermore, compared with the MOD group, the β-Gal activity in the SAL group decreased significantly (*p* < 0.05), suggesting that the SAL strain had a mitigating effect on this increase.

### 3.3 Effect of SAL strain intervention on oxidative stress levels in serum and liver of d-gal-induced aging mice

**Figure 2:**
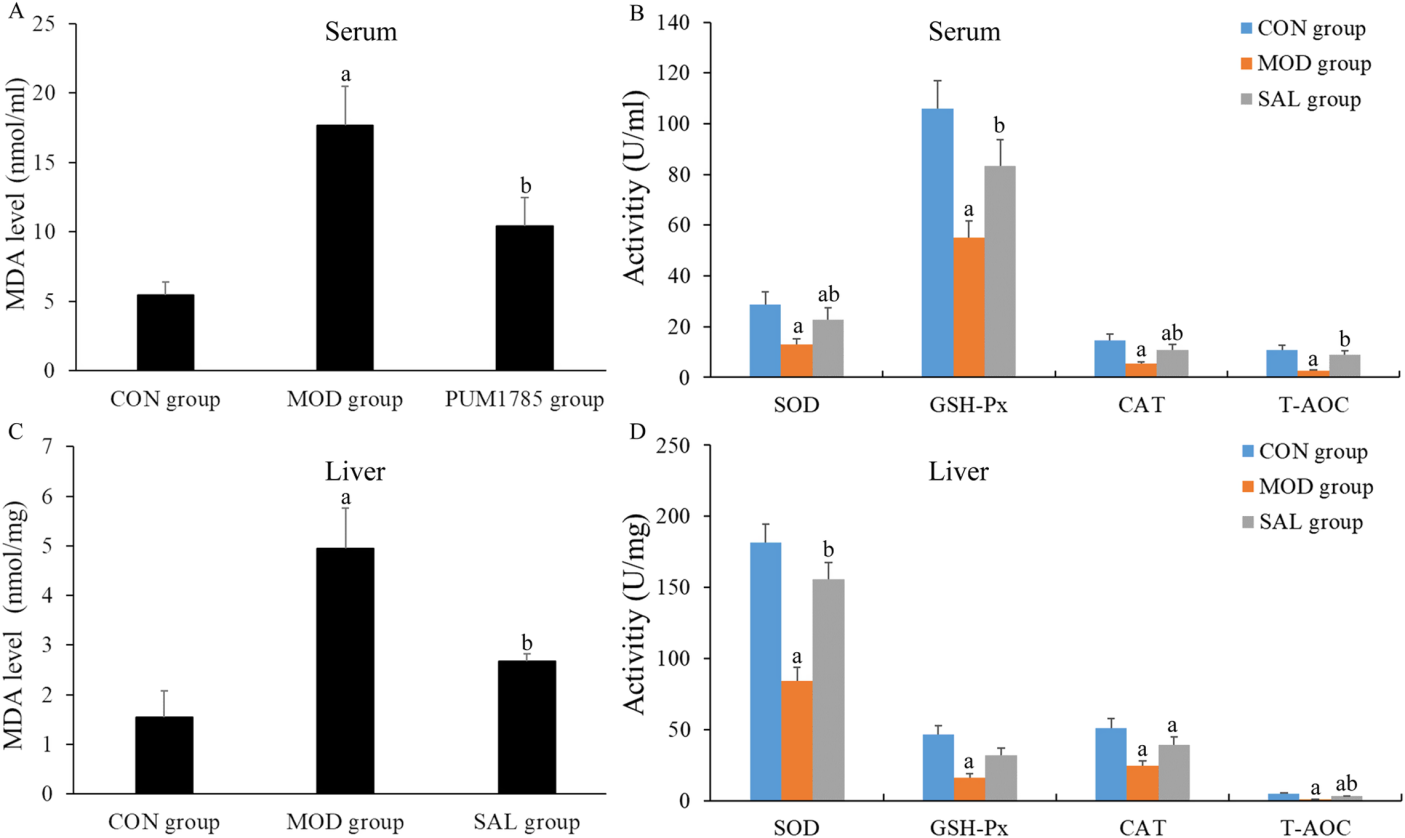
Effect of SAL strain intervention on the levels of oxidative stress indicators in serum and liver of d-gal-induced aging mice. **(A)** MDA level in serum; **(B)** SOD, GSH-Px, CAT, and T-AOC activities in serum. **(C)** MDA level in liver. **(D)** SOD, GSH-Px, CAT, and T-AOC activities in liver. a: Compared with the CON group, *p* < 0.05; b: compared with the MOD group, *p* < 0.05.

As shown in Figures 2A–D, compared with the CON group, the MOD group exhibited a significant increase in MDA levels in both serum and liver (*p* < 0.05), alongside a significant decrease in SOD, GSH-Px, CAT, and T-AOC activities in these tissues (*p* < 0.05). These findings suggest that oxidative stress occurred in the d-gal-induced aging mice, which is a hallmark of the aging process in mice. In contrast, compared with the MOD group, the SAL group showed a significant decrease in MDA levels in both serum and liver (*p* < 0.05). Moreover, there was a significant increase in SOD and T-AOC activities in these tissues (*p* < 0.05). Additionally, GSH-Px and CAT activities in the serum showed a significant increase (*p* < 0.05). However, although GSH-Px and CAT activities in the liver demonstrated an upward trend, the difference was not statistically significant (*p* > 0.05). These findings suggest that treatment with the SAL strain is capable of reversing the heightened oxidative stress levels induced by d-gal.

### 3.4 Effect of SAL strain intervention on intestinal barrier integrity of d-gal-induced aging mice

**Figure 3:**
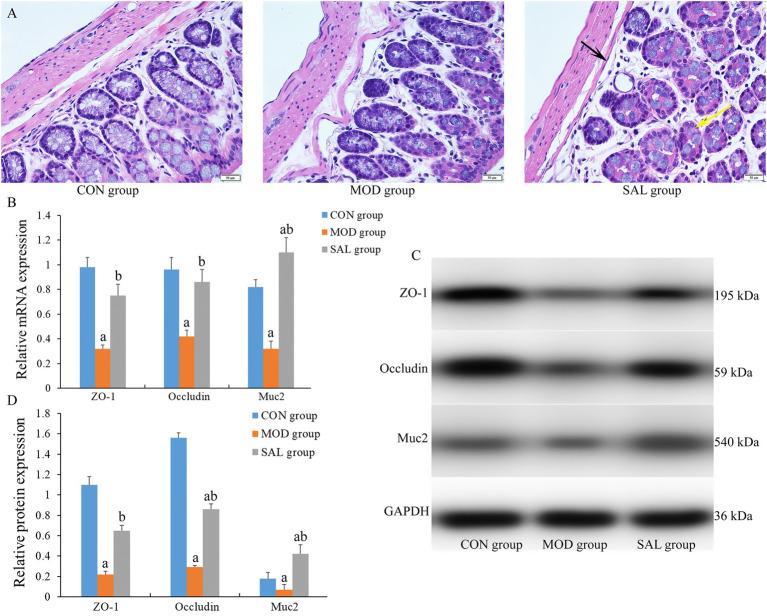
Effect of SAL strain intervention on colon histology of d-gal-induced aging mice. **(A)** Representative HE-stained sections of colon tissue (scale bars = 50 μm). Black arrow: narrow submucosal spaces with regular architecture; yellow arrow: diminished inflammatory cell infiltration. **(B)** mRNA expression levels of Zo-1, occludin and Muc2 genes in colon tissue. **(C)** Protein band images of Zo-1, occludin and Muc2. **(D)** Protein expression levels of Zo-1, occludin and Muc2 in colon tissue. a: Compared with the CON group, *p* < 0.05; b: compared with the MOD group, *p* < 0.05.

To evaluate the impact on the colon, histological analyses were performed using HE staining. As shown in Figure 3A, the CON group displayed normal colonic histology, featuring uniformly distributed glands and crypts, absence of inflammatory infiltrates, and a robust, continuous mucosal muscle layer. In contrast, the MOD group exhibited severe architectural disorganization characterized by mucosal layer damage, including glandular atrophy with crypt loss, diffuse inflammatory cell infiltration, marked thinning of the muscularis mucosa, and irregular submucosal expansion, collectively indicating compromised intestinal barrier function. The SAL group exhibited significant improvement, with restored crypt structures, an increased number of crypts, reduced inflammatory cells, and smaller, more regular submucosal spaces. These morphological improvements suggest the protective effects of the SAL strain intervention against d-gal-induced colonic injury.

Furthermore, as shown in Figures 3B–D, an analysis of the mRNA and protein expression levels of key tight junction proteins in the colon, specifically ZO-1, Occludin, and Muc2, is presented. The MOD group exhibited a significant decrease in both mRNA and protein expression levels of these proteins in the colon compared with the CON group (*p* < 0.05). In contrast, the SAL group exhibited a significant increase in these expression levels compared with the MOD group (*p* < 0.05). The administration of the SAL strain mitigated the d-gal-induced reduction in the expression levels of tight junction proteins.

### 3.5 Effect of SAL strain intervention on gut microbiota of d-gal-induced aging mice

#### 3.5.1 Species diversity

**Figure 4:**
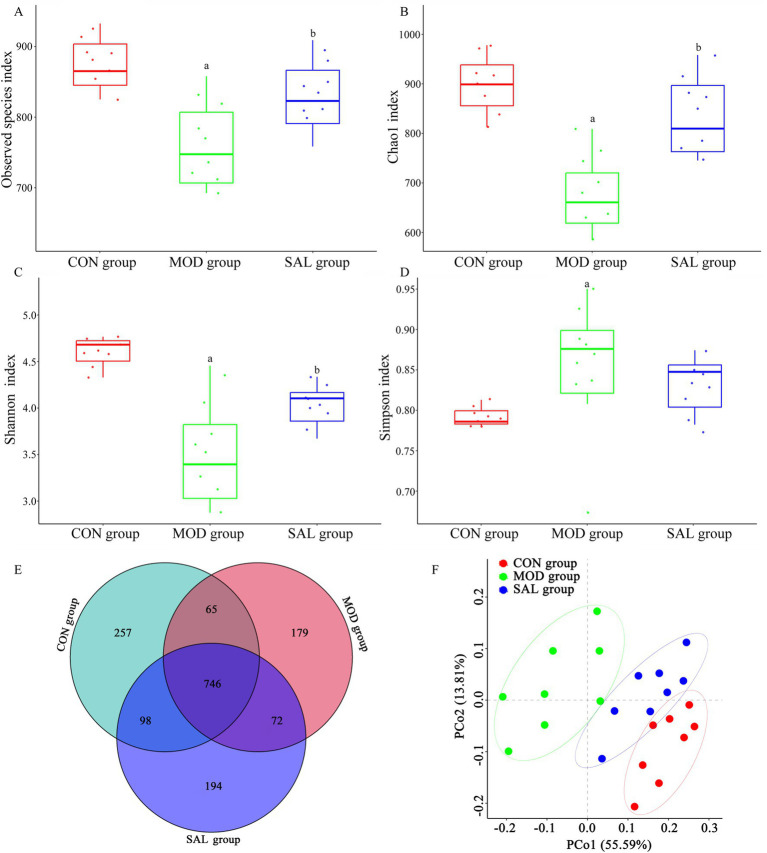
Effect of SAL strain intervention on gut microbiota diversity in d-gal-induced aging mice. **(A)** α-diversity presented as the index of Observed species. **(B)** α-diversity presented as the index of Chao1. **(C)** α-diversity presented as the index of Shannon. **(D)** α-diversity presented as the index of Simpson. **(E)** Venn diagram analysis. **(F)** β-diversity presented as PCoA. a: Compared with the CON group, *p* < 0.05; b: compared with the MOD group, *p* < 0.05.

The Observed species index, Chao1 index, Shannon index, and Simpson index were calculated to evaluate the α-diversity of the gut microbiota within each group. As shown in Figures 4A–D, compared with the CON group, the MOD group exhibited a significantly lower Observed species index, Chao1 index, and Shannon index, while it displayed a significantly higher Simpson index. In contrast, compared with the MOD group, the SAL group exhibited a significantly higher Observed species index, Chao1 index, and Shannon index. Although the Simpson index in the SAL group showed a downward trend, the difference was not statistically significant. The changes in α-diversity suggested that d-gal-induced aging in mice altered the gut microbiota richness, while Supplementation with the SAL strain, to some extent, restored microbial homeostasis, achieving partial recovery of phylogenetic diversity.

The Venn diagram visually represents the composition relationship of OTUs (operational taxonomic units) among samples within each group, serving as a means to assess the similarity and diversity of gut microbiota across groups. As shown in Figure 4E, there were distinct differences in OTU number composition among the three groups. A total of 1,611 OTUs were identified, with 746 shared OTUs representing the core microbiome (46.3% of the total community). The group-specific distributions were as follows: the CON group contained 1,166 OTUs, including 257 unique ones; the MOD group had 1,062 OTUs, with 179 unique ones; and the SAL group comprised 1,110 OTUs, of which 194 were unique. Notably, compared with the overlap between the CON and MOD groups (811 OTUs), the CON and SAL groups displayed slightly higher OTU conservation (844 shared OTUs), suggesting a potential protective effect of the SAL strain on microbiota composition.

PCoA was employed to visualize β-diversity patterns among different groups, with spatial distances between sample clusters quantitatively reflecting microbiota compositional similarity. In a PCoA plot, the closer the distance between samples, the higher the similarity in their microbiota composition; conversely, the farther the distance, the greater the difference. As shown in Figure 4F, PCoA revealed distinct β-diversity partitioning among the three groups, with the first two principal components explaining 55.59% (PC1) and 13.81% (PC2) of total variance, respectively. The three groups formed separate clusters, showing clear segregation and grouping among samples with minimal overlap and significant differences in microbiota structure among them (*p* < 0.05). Notably, spatial distribution patterns demonstrated maximal β-diversity divergence between the MOD and CON groups, indicating that d-gal induced intestinal flora disorder in mice. Moreover, samples from the SAL group tended to cluster nearer to the CON group, suggesting the SAL strain intervention might partially restore the structure of the gut microbiota to its normal state.

#### 3.5.2 Relative abundance of species at phylum and genus levels

**Figure 5:**
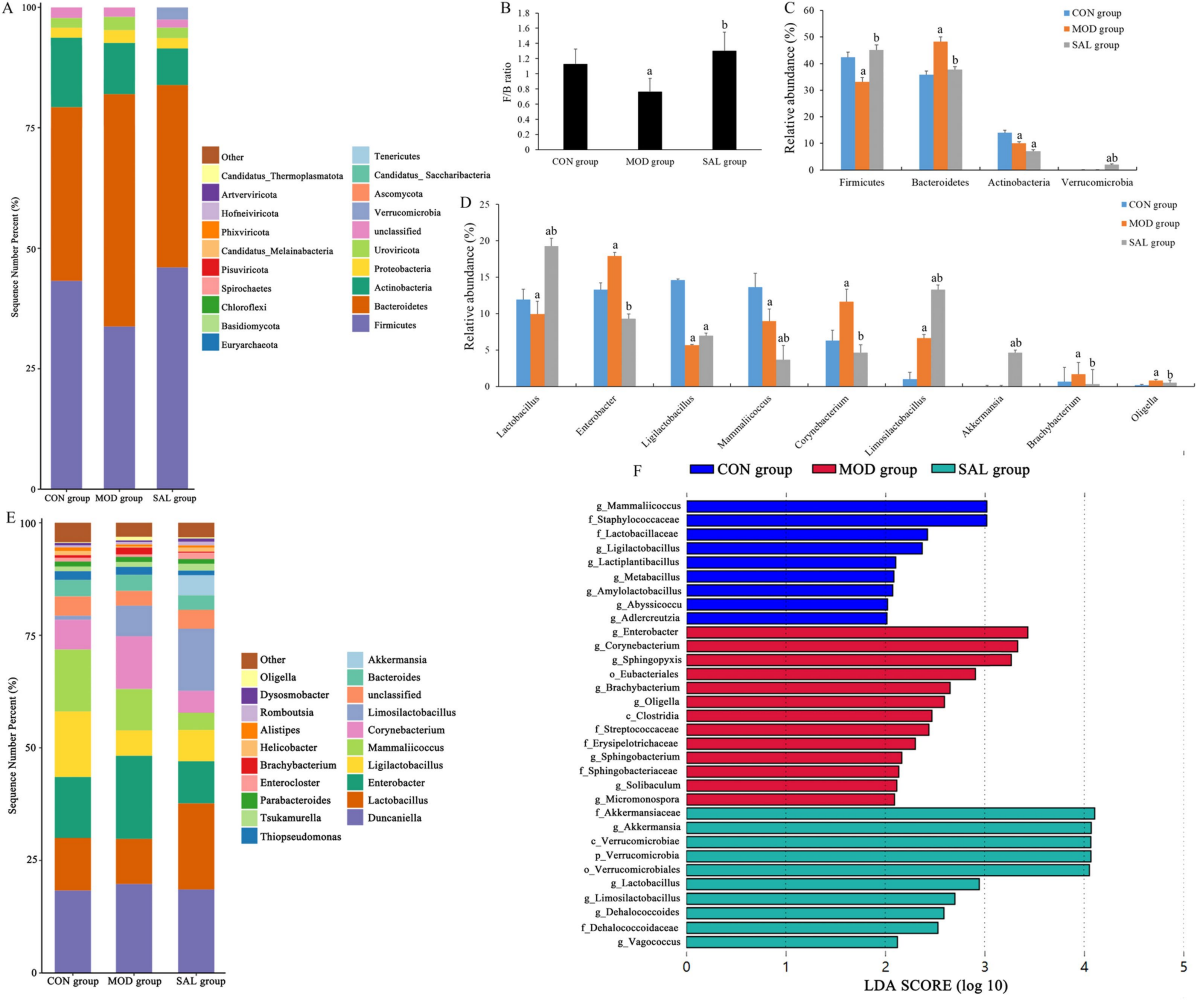
Effect of SAL strain intervention on gut microbiota structure in d-gal-induced aging mice. **(A)** Relative abundance of gut microbiota at phylum level. **(B)** The ratio of Firmicutes to Bacteroidetes. **(C)** Relative abundance of Firmicutes, Bacteroidetes, Actinobacteria, and Verrucomicrobia. **(D)** Relative abundance of *Lactobacillus*, *Enterobacter*, *Ligilactobacillus*, *Mammaliicoccus*, *Corynebacterium*, *Limosilactobacillus*, *Akkermansia*, *Brachybacterium*, and *Oligella*. **(E)** Relative abundance of gut microbiota at genus level. **(F)** LEfSe species difference analysis. a: Compared with the CON group, *p* < 0.05; b: compared with the MOD group, *p* < 0.05.

To further evaluate the function of probiotics, the colon contents of mice in each group were analyzed at both the phylum and genus levels. At the phylum level, the top 20 bacteria in each group were selected to draw the stacking bar chart. As shown in Figures 5A–C, Firmicutes, Bacteroidetes, and Actinobacteria were the dominant phyla among the three groups. It was clearly observed that the structure of the microbiota in each group was similar, yet the composition ratio differed. Compared with the CON group, the MOD group exhibited a decrease in the abundance of Firmicutes and Actinobacteria, while the abundance of Bacteroidetes increased, resulting in a significant decrease in the Firmicutes/Bacteroidetes (F/B) ratio (*p* < 0.05). In contrast, compared with the MOD group, the SAL group showed an increase in the abundance of Firmicutes and Verrucomicrobia, while the abundance of Bacteroidetes decreased, leading to a significant increase in the *F*/*B* ratio (*p* < 0.05).

Subsequently, a stacking bar chart was created to depict the top 20 bacteria at the genus level in each group. As shown in Figures 5D,E, the gut microbiota of each group was predominantly comprised of species such as *Duncaniella*, *Lactobacillus*, *Enterobacter*, *Ligilactobacillus*, *Mammaliicoccus*, *Corynebacterium*, *Limosilactobacillus*, and *Bacteroides*. Compared with the CON group, the MOD group exhibited a decrease in the abundance of *Lactobacillus*, *Ligilactobacillus*, and *Mammaliicoccus*, whereas an increase in the abundance of *Enterobacter*, *Corynebacterium*, *Brachybacterium*, and *Oligella*. Compared with the MOD group, the SAL group demonstrated an increase in the abundance of *Lactobacillus*, *Ligilactobacillus*, *Limosilactobacillus*, *Akkermansia*, *Tsukamurella*, and *Enterocloster,* whereas a decrease in the abundance of *Enterobacter*, *Mammaliicoccus*, *Corynebacterium*, *Bacteroides*, *Thiopseudomonas*, *Brachybacterium*, and *Oligella.*

#### 3.5.3 Intergroup species differences

To gain a deeper understanding of the intestinal flora types and identify the specific bacteria that characterized each group, LEfSe (Linear Discriminant Analysis Effect Size) analysis was employed. This method identified distinct phylogenetic features representing the microbiota composition of each group, with LDA values > 2 regarded as indicating statistically significant differences among groups. As shown in Figure 5F, there was a total of 21 discriminative features in the three groups. Specifically, one significantly different phylum was identified, which was Verrucomicrobia, and it was enriched in the SAL group. Moreover, there were 20 significantly different genera. Among these, 7 were present in the CON group, including *Mammaliicoccus*, *Ligilactobacillus*, *Lactiplantibacillus*, *Metabacillus*, *Amylolactobacillus*, *Abyssicoccus*, and *Adlercreutzia*. Eight were identified in the MOD group, namely *Enterobacter*, *Corynebacterium*, *Sphingopyxis*, *Brachybacterium*, *Oligella*, *Sphingobacterium*, *Solibaculum*, and *Micromonospora*. Lastly, 5 were detected in the SAL group, including *Akkermansia*, *Lactobacillus*, *Limosilactobacillus*, *Dehalococcoides*, and *Vagococcus*.

### 3.6 Correlation between the gut microbiota composition and antioxidant indices in d-gal-induced aging mice

**Figure 6:**
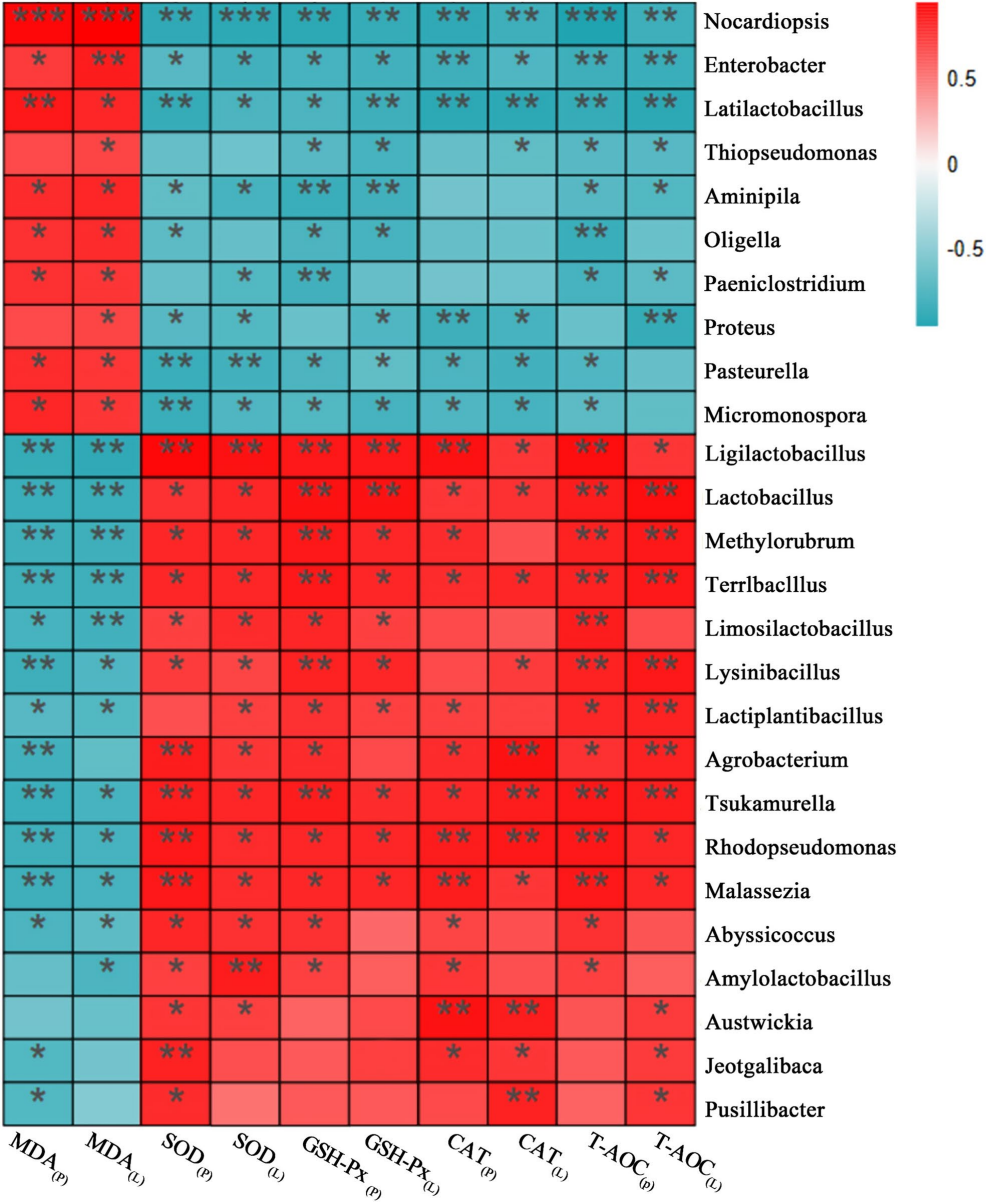
The correlation heatmaps of gut microbiota with antioxidant indices in d-gal-induced aging mice. *p < 0.05, **p < 0.01, ***p < 0.001.

To elucidate potential microbiota-host interactions, Spearman correlation analysis was performed at the genus level to evaluate the relationships between gut microbial composition and systemic oxidative stress parameters. A comprehensive correlation heatmap was generated to visualize significant associations between discriminant bacterial genera and key oxidative stress biochemical indicators, including MDA, SOD, GSH-Px, CAT, and T-AOC in both serum and liver tissue. As depicted in Figure 6, the relative abundance of significantly differential genera in the CON group, including *Ligilactobacillus*, *Lactiplantibacillus*, *Amylolactobacillus*, and *Abyssicoccus*, was significantly negatively correlated with MDA content in serum and liver tissue (*p* < 0.05) and significantly positively correlated with SOD, GSH-Px, CAT, and T-AOC activities (*p* < 0.05). In the MOD group, the significantly differential genera exhibited pro-oxidative profiles. Specifically, *Enterobacter*, *Oligella*, and *Micromonospora* were significantly positively correlated with MDA content (*p* < 0.05). Moreover, *Enterobacter* and *Micromonospora* were significantly negatively correlated with SOD, GSH-Px, CAT, and T-AOC activities (*p* < 0.05), while *Oligella* was significantly negatively correlated with SOD, GSH-Px, and T-AOC activities (*p* < 0.05). In the SAL group, the significantly differential genera, including *Lactobacillus* and *Limosilactobacillus*, demonstrated strong antioxidant potential. These genera exhibited negative correlations with MDA content (*p* < 0.05) and positive correlations with antioxidant activities. Notably, *Lactobacillus* was significantly positively correlated with SOD, GSH-Px, CAT, and T-AOC activities (*p* < 0.05), while *Limosilactobacillus* was significantly positively correlated with SOD, GSH-Px, and T-AOC activities (*p* < 0.05).

## 4 Discussion

With the continuously growing global aging population, health problems related to aging and associated diseases have become increasingly significant. Effectively delaying the aging process and enhancing the health of the elderly have become top priorities in life science research. Studies show that oxidative stress and gut microbiota dysbiosis play crucial roles in the development and progression of aging and its related diseases (Tanriover et al., 2023; Wu et al., 2021). Probiotics can exert various beneficial functions in the body, such as enhancing antioxidant capacity to eliminate accumulated free radicals, modulating gut microbiota balance by increasing beneficial bacterial abundance and suppressing pathogenic species, thereby achieving the effects of delaying aging and protecting health. Research indicates that certain *Lactobacillus* strains exhibit colonization potential in the gut, contributing to the improvement of host health through sustained probiotic effects (Lan et al., 2022; Noureen et al., 2023). *Lactobacillus plantarum* SAL was isolated from the feces of patients who had undergone long-term antibiotic or hormone therapy, where common probiotics struggle to colonize, suggesting that SAL strain may possess strong tolerance and adhesion capabilities, demonstrating exceptional environmental adaptability. Preliminary *in vitro* experiments confirmed that the SAL strain has a high survival rate under artificial gastric juice and bile acid stress conditions, as well as antibacterial and antioxidant properties, highlighting its probiotic potential. This study demonstrates through animal experiments that the SAL strain improves oxidative stress status and gut microbiota structure in d-gal-induced aging mice, achieving significant anti-aging effects.

### 4.1 The SAL strain alleviates age-related oxidative damage

Intraperitoneal injection of d-gal serves as a major approach for establishing aging animal models (Azman and Zakaria, 2019; Kumar H. et al., 2022). Prolonged administration of d-gal induces excessive generation of reactive oxygen species (ROS) such as H_2_O_2_ and O_2_^−^, which disrupts the endogenous antioxidant enzyme system comprising SOD, GSH-Px, and CAT. This oxidative imbalance leads to diminished T-AOC, metabolic dysregulation of free radicals, and oxidative damage to critical biomolecules including lipids, proteins, and nucleic acids. Consequently, the structural integrity of cells and tissues is compromised, affecting normal growth and development, accelerating senescence, and manifesting premature aging phenotypes that mimic natural aging processes (Kumar H. et al., 2022). In this study, mice received daily intraperitoneal injections of 10% d-gal solution (300 mg/kg body weight) for 10 consecutive weeks. The results demonstrated that d-gal intervention resulted in growth retardation (evidenced by suppressed weight gain), elevated β-Gal activity (a senescence biomarker in hepatic cells), and significant oxidative stress markers, including decreased activities of SOD, GSH-Px, CAT, and T-AOC alongside increased MDA levels in both serum and liver tissue. These findings align with previous modeling studies, indicating the successful establishment of the d-gal-induced aging mouse model (Gao et al., 2022; Jin et al., 2024). Notably, following 10 weeks of SAL strain intervention, the growth of the model mice was significantly improved, as evidenced by an increase in body weight, enhanced activities of SOD, GSH-Px, CAT, and T-AOC, reduced MDA levels in both serum and liver tissue, and a marked decrease in β-Gal activity in liver tissue. These effects reversed oxidative stress induced by aging, consistent with outcomes from other probiotic intervention studies (Kumar H. et al., 2022; Shen et al., 2022). The SAL strain possesses antioxidant properties, and its conserved genes encoding antioxidant proteins, such as *purA* (encoding adenylosuccinate synthetase) and *gadB* (encoding glutamate decarboxylase), may serve as triggers for activating the antioxidant enzyme system. This activation results in stabilizing lipid membranes against peroxidation and preserving the integrity of subcellular organelles. Consequently, the SAL strain effectively alleviates age-related oxidative damage. This evidence highlights its potential as a therapeutic candidate for anti-aging and age-related diseases.

### 4.2 The SAL strain alleviates age-related intestinal structural damage and restores microbial homeostasis

Aging begins in the gut (Escudero-Bautista et al., 2024; Sharma, 2022). With advancing age, excessive free radicals accelerate the senescence of intestinal epithelial cells, leading to structural alterations in the intestinal tissue. These changes manifest as reduced number and shortened length of intestinal villi, decreased crypts and goblet cells, thinning of the intestinal mucosa, and diminished levels of tight junction proteins (e.g., ZO-1, Occludin) and mucin proteins (e.g., Muc2) (Chen et al., 2024; Choi et al., 2024). These degenerative processes increase intestinal permeability and compromise barrier function, thereby accelerating aging and promoting age-related diseases. ZO-1 and Occludin, key intestinal epithelial tight junction proteins, play central roles in maintaining the structural integrity of the intestinal physical barrier (Kuo et al., 2022). Muc2, synthesized and secreted by abundant goblet cells in colonic crypts, constitutes a critical component of the intestinal chemical barrier (Zhang et al., 2024). Beyond directly preserving intestinal barrier integrity, ZO-1, Occludin, and Muc2 are indispensable for protecting the intestine and maintaining its homeostasis (Duan et al., 2023). Studies have shown that gut microbiota homeostasis at the phylum level is dominated by Firmicutes and Bacteroidetes (Banaszak et al., 2023; Jandhyala et al., 2015). During the aging process, the abundance of Firmicutes declines, whereas that of Bacteroidetes increases, resulting in a decreased F/B ratio, which serves as an indirect indicator for assessing gut health and the progression of aging (Gao et al., 2021; Wang et al., 2020). In this study, the d-gal-induced aging model (MOD group) exhibited age-related intestinal alterations, including structural damage to the mucosal layer, atrophy of glands and crypts, thinning of the muscularis mucosae, irregular submucosal expansion, and downregulated mRNA and protein levels of ZO-1, Occludin, and Muc2. Venn diagrams and PCoA analysis revealed significant microbiota compositional shifts in the MOD group compared with the CON group, characterized by reduced Firmicutes and Actinobacteria, increased Bacteroidetes, and a lowered F/B ratio. These findings confirm d-gal-induced intestinal aging, structural barrier damage, and functional impairment, further validating the success of the aging model.

An intact intestinal barrier and stable gut microbiota are essential prerequisites for maintaining intestinal and systemic health. Certain probiotics have been shown to restore intestinal and systemic health by repairing gut structure and modulating microbial diversity, thereby delaying aging processes and mitigating the risk of age-related diseases (Jin et al., 2024; Zhao et al., 2018). In this study, the SAL strain intervention significantly improved intestinal morphology of mice, as evidenced by increased colonic crypt numbers with restored architecture, reduced inflammatory cell infiltration, narrowed and regularized submucosal spaces, and elevated mRNA and protein levels of ZO-1, Occludin, and Muc2. Gut microbiota analysis revealed that total and unique OTU counts in the SAL group closely resembled to those in the CON group, with the flora diversity patterns also aligning more closely with those in the CON group. Notably, the SAL strain increased the abundance of Firmicutes and Verrucomicrobia while decreasing the abundance of Bacteroidetes, resulting in an elevated F/B ratio. These findings suggest that the SAL strain, akin to other *L. plantarum* strains, enhances intestinal tight junctions and mucus secretion, reverses microbial composition shifts at the phylum level (especially enriching Verrucomicrobia), and restores gut microbiota homeostasis (Cheng et al., 2020; Jeong et al., 2015; Kumar A. et al., 2022; Zeng et al., 2023). This intervention alleviates aging-induced structural and barrier dysfunction in the gut, thereby exerting protective effects on intestinal health. Studies indicate that age-related dysbiosis is often accompanied by reduced Verrucomicrobia abundance, which weakens the suppression of pathogenic bacteria and impairs the integrity of the intestinal barrier (Alam et al., 2021). *Lactobacillus plantarum* produces short-chain fatty acids (SCFAs) and antimicrobial metabolites during intestinal fermentation. These compounds inhibit pathogenic bacteria, increase mucosal thickness, and elevate mucin levels, and create a gut microenvironment favorable for colonization and growth of bacteria belonging to the Verrucomicrobia phylum, such as Akkermansia. These findings underscore the role of the SAL strain in mitigating age-related intestinal structural damage and microbial dysregulation, which is consistent with the mechanisms employed by other *L. plantarum* in promoting gut health (Liu et al., 2020; Wang et al., 2021).

### 4.3 The correlation between gut microbiota composition and individual oxidative status

Previous studies have confirmed a close association between gut microbial composition and individual oxidative status (Kunst et al., 2023; Riaz Rajoka et al., 2021). In this study, LEfSe analysis and Spearman correlation analysis were conducted at the genus level to further investigate the relationship between the significantly different genera in each group and antioxidant indicators, in order to better understand the gut microbiota-mediated mechanism of the SAL strain in alleviating oxidative stress *in vivo*. Results revealed that host-associated genera *Lactobacillus*, *Lactiplantibacillus*, *Amylolactobacillus, Abyssicoccus*, and *Limosilactobacillus* exhibited positive correlations with antioxidant capacity during aging, whereas *Enterobacter*, *Oligella*, and *Micromonospora* showed negative correlations. Notably, *Enterobacter* and *Oligella*, classified as potential pathobionts, lack conventional antioxidant enzyme defense systems, which impairs their ability to scavenge free radicals (Liu et al., 2019; Lu et al., 2022). The antioxidant efficacy of the SAL strain may primarily be attributed to its significant enrichment of *Lactobacillus* and *Limosilactobacillus* within the Firmicutes phylum. The proliferation of these two genera may be linked to SAL strain’s survival and colonization in the gut. *Lactobacillus* exerts antioxidant effects through multiple pathways, including modulation of the AMPK/SIRT1 and Keap1/Nrf2 signaling pathways, which are key regulators of oxidative stress-related protein activation, as well as the production of SCFAs, which are crucial for maintaining redox homeostasis (Dong et al., 2024; Li et al., 2021; Li et al., 2024; Wang et al., 2021). This integrated mechanism highlights the SAL strain’s potential in reshaping gut microbiota composition to counteract age-related oxidative stress.

## 5 Conclusion

In summary, the d-gal-induced aging model is associated with oxidative stress damage and structural-functional dysbiosis of the gut microbiota. Intervention with *L. plantarum* SAL ameliorates these aging-related phenotypes by modulating microbial composition, notably enriching the abundance of beneficial bacteria and reducing the pathogenic bacteria. These microbial shifts contribute to the protection of intestinal structure and function, alleviate systemic oxidative stress injury, and ultimately achieve the purpose of improving aging. On this ground, *L. plantarum* SAL, a safe probiotic strain with anti-aging potential, could be used as a potential functional food to delay aging and prevent age-related diseases.

## Data Availability

The datasets presented in this study can be found in online repositories. The names of the repository/repositories and accession number(s) can be found in the article/Supplementary material.
