# Supplementary material for: Lactobacillus plantarum SAL delays aging-associated oxidative stress and gut microbiota dysbiosis in mice
Source: Front Microbiol. 2025 Jun 16;16:1607824. doi: 10.3389/fmicb.2025.1607824 (PMC12206855; doi:10.3389/fmicb.2025.1607824)
Supplement: Supplementary file 1 [file Table_1.doc]

Table 1. Oligonucleotide primers used for qRT-PCR.

| Gene | Forward | | Reverse | |  |
| --- | --- | --- | --- | --- | --- |
| *ZO-1* | | GCTGGAGAAGATGGAGAA | | CAGGTCCTCCTGGTCTTCTC | |
| *Occludin* | | CCTCTGGCT TTGCTTCTGTC | | TGAGGATGGTGCTGAGTTTG | |
| *Muc2* | | GCTGCTCCTGCTGCTACTAC | | CAGGTCATCGTCATCGTCTC | |
| *GAPDH* | | AGGTCGGTGTGAACGGATTTG | | TGTAGACCATGTAGTTGAGGTCA | |
